# Nanogold-coated stent facilitated non-invasive photothermal ablation of stent thrombosis and restoration of blood flow†

**DOI:** 10.1039/d3na00751k

**Published:** 2024-01-30

**Authors:** Nitesh Singh, Paresh P. Kulkarni, Prashant Tripathi, Vikas Agarwal, Debabrata Dash

**Affiliations:** a Centre for Advanced Research on Platelet Signaling and Thrombosis Biology, Department of Biochemistry, Institute of Medical Sciences, Banaras Hindu University Varanasi-221005 India ddash.biochem@gmail.com niteshsingh264@gmail.com pareshkulbmc@gmail.com; b School of Physical Sciences, Jawaharlal Nehru University New Mehrauli Road New Delhi Delhi-110067 India pra.jest01@gmail.com; c Department of Cardiology, Institute of Medical Sciences, Banaras Hindu University Varanasi-221005 India vikky25@yahoo.com

## Abstract

In-stent restenosis (ISR) and stent thrombosis (ST) are the most serious complications of coronary angioplasty and stenting. Although the evolution of drug-eluting stents (DES) has significantly restricted the incidence of ISR, they are associated with an enhanced risk of ST. In the present study, we explore the photothermal ablation of a thrombus using a nano-enhanced thermogenic stent (NETS) as a modality for revascularization following ST. The photothermal activity of NETS, fabricated by coating bare metal stents with gold nanorods generating a thin plasmonic film of gold, was found to be effective in rarefying clots formed within the stent lumen in various *in vitro* assays including those under conditions mimicking blood flow. NETS implanted in the rat common carotid artery generated heat following exposure to a NIR-laser that led to effective restoration of blood flow within the occluded vessel in a model of ferric chloride-induced thrombosis. Our results present a proof-of-concept for a novel photothermal ablation approach by employing coated stents in the non-invasive management of ST.

## Abstract 2

The schematic representation illustrates deployment of a nanogold-coated stent for achieving non-invasive photothermal ablation of stent thrombosis upon NIR laser irradiation, leading to the restoration of blood flow.

## Graphical abstract

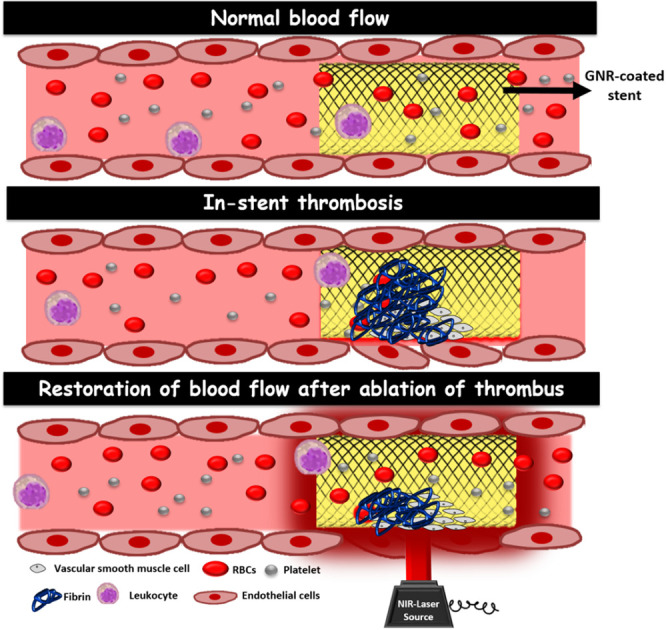

## Introduction

Coronary artery disease (CAD) is the leading cause of mortality worldwide with an estimated 17.9 million people succumbing to it each year.^1,2^ Ever since the first successful coronary artery bypass procedure was performed by Rene Favaloro in 1968, it has become a standard of care in patients with significant coronary atherosclerosis. However, due to this being a major surgery and a highly invasive procedure, angioplasty was developed as a relatively non-invasive substitute. Although balloon angioplasty was effective in the management of coronary stenosis due to atherosclerotic plaque, its use was associated with serious complications. Stents were developed in an attempt to provide a temporary scaffold to tide over limitations associated with balloon angioplasty. Stent implantation eliminated acute vessel recoil and chronic constrictive remodelling, thereby reducing restenosis rates. Use of adjuvant dual antiplatelet therapy also reduced the rates of thrombosis to acceptable levels. In sum, current treatment options for CAD include anti-platelet medications, percutaneous coronary intervention (PCI) and coronary artery bypass grafting (CABG). PCI with stenting has become the preferred mode of treatment, especially when the extent of stenosis is more than 50%. Although highly effective, the procedure is plagued with two serious limitations.^1–3^ First, there could be thrombosis in the peri-stent region (stent thrombosis or ST), which can occur early (less than 30 days), late (30 days to 1 year) or very late (more than 1 year) following the procedure. The common etiological mechanisms of ST include: (i) exposure of blood to the prothrombotic subendothelial matrix, stent struts and/or polymer before they are covered by re-endothelialization, (ii) persistent slow coronary blood flow, (iii) inadequate suppression of platelet activation by antiplatelet drugs and (iv) systemic prothrombotic states.^4^ The rates of ST vary from as high as 20% without antiplatelet/anticoagulant drugs^5^ to as low as 2% with aggressive dual anti-platelet therapy.^6^ Second, there could be re-narrowing of the vessel lumen (in-stent restenosis or ISR) in 10–30% of the patients^7–9^ characterized by excessive smooth muscle cell proliferation and migration into the intimal layer. This complication of neointimal hyperplasia is addressed with drug-eluting stents (DES), which release anti-proliferative medications like sirolimus and paclitaxel. Although DES remarkably restricted the incidence of ISR to less than 10%, they are ironically associated with a higher risk of late or very late ST than bare metal stents (BMS) due to several reasons.^4^ First, re-endothelialization is significantly delayed with DES compared to BMS, thus providing a substrate of injured vessel wall for eventual stent thrombosis.^10^ Second, late stent malapposition as a result of positive vessel wall remodelling away from the stent is more common with DES than BMS.^11^ Lastly, appearance of new atherosclerotic plaques in the peri-stent area, whose rupture can lead to ST, occurs earlier in DES than BMS.^12,13^ ST is life-threatening with mortality rates ranging from 11% to 42% and warrants immediate repeat angioplasty or CABG surgery. However, it is important here to emphasize that there has been no development in coronary stent technologies to directly address this complication.

Advantages of minimal invasive photothermal therapy have widely been recognized as an effective, safer and sound approach in wound healing,^14–17^ treatment of bacterial infection,^18–21^ pain relief,^22,23^ drug delivery^24–27^ and cancer management.^27–29^ For example, an attempt has been made to manage obstructive rectal cancer^30^ or minimize rat esophageal granulation tissue^31^ with heat generated from photothermally active stents. By employing both *in vitro* assays and a murine model of thrombosis *in vivo*, we have for the first time demonstrated that fibrin clots/thrombi could be destabilized by heat due to dissociation of non-covalently assembled fibrin monomers, and provided a proof-of-concept to harness photothermal therapy as an effective tool towards targeted thrombolysis.^32^ Our study was succeeded by several follow-up reports supporting the hypothesis.^33–35^ The concept introduced a safe and smart approach to revascularization, which minimizes the off-target life-threatening complications associated with the currently employed fibrinolytic drug regime. In the present study, we have extended this concept to fabricate novel thermogenic stents employing GNRs, which have the ability to generate heat upon irradiation with a NIR-laser from outside. Thermogenic stents implanted *in situ* can effectively bring about photothermal lysis of in-stent/peri-stent clots that can be washed off from lesion sites by arterial hydrodynamic shear, leading to partial or complete restoration of blood flow. This being the first study would need further investigation towards clinical validation.

## Materials and methods

Alexa Fluor 488 – conjugated fibrinogen was purchased from Invitrogen. Human fibrinogen, thrombin and gold nanorods (GNRs) (axial diameter: 9.0–11.0 nm; longitudinal diameter: 36.9–45.1 nm), methyl methacrylate (MMA), butylmethacrylate (BMA), methylbenzoate, polyethylene glycol 400, benzoyl peroxide and *N*,*N*-dimethyl-*p*-toluidine were procured from Sigma, while methylene blue, benzoyl peroxide and Drabkin's solution were products of Merck, Thomas baker and Linear Chemicals S.L.U., respectively. The rest of the chemicals were either from Sigma or Merck. All reagents were of analytical grade. Type I deionized water (18.2 MΩ cm, Millipore) has been used throughout the experiment.

### Stents

For *in vitro* studies bare metal cobalt–chromium alloy stents (Flexinnium, length, 32 mm; diameter, 2 mm; strut thickness, 60 μm) were procured from M/S Sahajanad Medical Technology, India. For implantation in the rat carotid artery stainless steel stents (length, 2.5 mm; diameter, 635 μm; strut thickness, 70 μm) were purchased from M/S Olexander R&D Inc., USA.

### Thin-layer coating of NIR-active nanomaterials on the stent surface

Thin layer coating of gold on the bare metal stent surface by employing photothermally active gold nanorods was achieved in three steps: (a) activation step: prior to coating, the stent surface was activated by treatment with HCl (0.1 M) solution for 1–2 h. This was followed by washing with type I deionized water and short bath sonication (Branson 2510 Ultrasonic Bath, 40 Hz) to remove excess acid. The washing was repeated 3 times and the stent was dried in a vacuum oven (Anil Scientico) at 80 °C for 20 min. (b) Coating step: next, assembly of the nanomaterial on the stent surface was accomplished by bath sonicating the stent in the presence of gold nanorods (30 μg ml^−1^) for 10 min each time with 10 min intervals for up to 3 h, which was followed by stirring in GNR solution with an Eppendorf Thermomixer (model 5350) for 4 h. (c) Annealing step: finally, the thin plasmonic film of gold on the stent was achieved by placing the stent in a vacuum oven at 350 °C under an inert (nitrogen gas) environment for 3 h. The coating and annealing steps were repeated for 4 cycles to achieve a dense layer of gold coating on the stent surface.

### Characterization of nano-enhanced thermogenic stents (NETS)

Surface morphologies of stents following coating were characterized by scanning electron microscopy (SEM) and energy-dispersive X-ray spectroscopy (EDS or EDX) coupled with SEM (M/S FEI quanta 200). The bare metal and coated stents were mounted on carbon tape adhered on a sample holder (stub) and SEM and EDX images were captured.^36^ The stent underwent additional characterization through a comprehensive analytical approach utilizing Raman spectroscopy and Fourier-transform infrared spectroscopy (FTIR) in attenuated total reflection (ATR) mode (detailed information in the ESI section†).

The photothermal efficiency of NETS was evaluated by irradiating the stents with a 3 W solid-state continuous diode laser (808 nm) (M/S Changchun New Industries, China) at 1.05 W cm^−2^ and the rise in temperature was recorded with an infra-red thermal camera (M/S FLIR, USA) (ESI Fig. 1†). In order to investigate the stability of the coating on the stent surface when exposed to hydrodynamic shear of perpetual blood flow as may occur within the coronary artery, a buffer was allowed to flow over the coated stents employing a high-resolution peristaltic pump (Bio-Rad). The superfusion of the buffer upon the stent was maintained consistently for 6 to 8 h each day for 30 days. The stability of the coating was then evaluated from the uniformity in heat generated on different days when the stent was irradiated with a laser.

### Characterization of thrombolysis elicited in and around NIR laser-irradiated NETS *in vitro*

Clot formation (fibrin polymerization) was induced around the thermogenic stent by addition of 2–5 mM CaCl_2_ and 1 U ml^−1^ thrombin to purified human fibrinogen as described.^32^ Whenever required, a fluorescent fibrin clot was generated by adding 45 μl purified fibrinogen (1 mg ml^−1^) and 5 μl Alexa Fluor 488 – conjugated fibrinogen. Next, the stent was irradiated with an NIR laser and the extent of clot lysis due to the heat generated from the fabricated stents was determined by various assays as already reported.^32^ (a) Fluorescence-based assay: NETS was irradiated with a NIR laser for 30 min and the supernatant was collected. The presence of fluorescent fibrin in the supernatant was analysed with a fluorescence multi-mode microplate reader (BioTek model Synergy H1) (excitation, 488 nm; emission, 519 nm) as a measure of fibrin released due to thrombolysis. (b) Drabkin's assay: a clot containing trapped RBCs was generated around the stent by mixing 2.5 μl RBCs and 47.5 μl fibrinogen solution followed by thrombin and CaCl_2_ as mentioned above. Thermal disruption of the thrombus released RBCs, which was quantified using Drabkin's reagent. The supernatant (containing released RBCs) was added to an equal volume of Drabkin's solution (K_2_HPO_4_, 50 mM; KCN, 38 mM; [K_3_Fe(CN)_6_], 30 mM; surfactant, 2–5% w/v), and kept at RT for 20 min. Absorbance was recorded at 540 nm as a measure of the cyanoderivative of hemoglobin. (c) Methylene blue assay: ablation of the clot was determined from the extent of release of methylene blue from fibrin mesh around the stent. The clot was induced to form as described above where 0–1 mg ml^−1^ dye replaced the RBCs. Following laser irradiation of the NETS the supernatant was collected, and the extent of methylene blue released was recorded from the absorbance at 540 nm. (d) Scanning electron microscopy (SEM): the change in surface morphology of the fibrin clot and thrombus-depleted areas, as well as diameter of fibrin fibres was recorded using an analytical SEM^37,38^ as mentioned in the ESI section.† (e) Confocal laser scanning microscopy (CLSM): the fiber density and thrombus-depleted areas in the fluorescently labelled clots were analysed employing confocal microscopy^37,38^ (details in the ESI section†).

### Fluorescence recovery after photobleaching (FRAP) analysis

The altered molecular dynamism within the structure of the in-stent/peri-stent thrombus following exposure to the NIR laser, reflective of fibrin depolymerization/repolymerization kinetics, was evaluated employing the FRAP analysis tool. A fluorescent clot was generated around the stent as stated above. The clot-moulded stent was carefully transferred to a fabricated microscopic slide (bordered with adherent tape as a spacer; depth: 450 μm) as already described.^32,39^ The in-stent clot was exposed to the NIR laser (808 nm) in a humid chamber for 15 min. FRAP was performed on a confocal microscope (Zeiss, model LSM 700) equipped with a plan-apochromat 1.4 NA oil immersion objective (63×), 488 nm argon ion laser, and LSM acquisition software. The detector gain and offset were set before collecting FRAP images. The detector gain was set to a level such that very few or no pixels were saturated. Selected areas on the fluorescent thrombus were photobleached using the 488 nm laser at 100% output at a rate of 20 ms per pixel. Five pre-bleached images were captured as a reference for the steady-state distribution of fluorescent molecules. Images of fluorescence recovery were collected at 2% excitation laser power using Zen Imaging software and analysed using ImageJ software (National Institutes of Health). Data were analysed with GraphPad Prism software.

### NETS-mediated thrombolysis under arterial shear

In order to evaluate stent thrombolysis against arterial fluid shear resembling the physiological milieu, a fluorescent thrombus was generated around the stent as described above. The stent carrying the thrombus was placed within a fabricated silicon tube, and fluid was allowed to perfuse over it at arteriolar hydrodynamic shear (1500 s^−1^) precisely regulated with a high-resolution peristaltic pump (Bio-Rad) (ESI Fig. 2†).^32^ The stent was exposed to the NIR laser for 30 min and the extent of thrombolysis was quantified from the release of the fluorescent fibrin in the fluid of the reservoir as described above.

### In-stent/peri-stent thrombolysis by the heat generated from a NIR laser-irradiated thermogenic stent grafted in the rat common carotid artery *in situ*

#### (a) Stent angioplasty

Stent implantation in the murine common carotid artery was carried out as described^40,41^ with minor modifications. Briefly, Swiss albino adult rats (250–300 g) (*n* = 14) were administered aspirin (10 mg kg^−1^ d^−1^) orally for 5 days.^42^ Rats were anesthetized using 3% isoflurane strictly as per recommendations of the Animal Ethical Committee of Banaras Hindu University. After careful shaving and proper disinfection of the area a small median incision was made in the ventral neck region with standard surgical tools to isolate the common carotid artery of the rats along with external and internal carotids under a stereo microscope (Zeiss Stemi 2000; magnification: 6.5–50×). The external carotid artery was permanently ligated as distal as possible using a 5/0 silk thread (Merilsilk). The proximal parts of the common and internal carotid arteries were temporarily occluded by binding knots with the 5/0 silk thread to interrupt blood flow (Fig. 6). The exposed vessels were fixed or stretched in such a way that the common and external carotid arteries fell in a straight line, which allowed easy stent deployment. A small incision was made in the external carotid artery. The coated stainless steel thermogenic stent (length, 2.5 mm; diameter, 635 μm; strut thickness, 70 μm) was carefully introduced through arteriotomy proximal to ligation, which was further guided to the common carotid artery using a silicone tube. After the stent reached the desired location, the silicon tube was pulled back and the stent was allowed to inflate within the artery. Next, the temporary sutures were removed from the common and internal carotid arteries to allow blood flow along the implanted NETS.

#### (b) Photoacoustic (PA) imaging of the implanted stent

The stent was implanted at the desired location in the anesthetized rat after the surgical procedure described above. The animal was then placed on a Vevo imaging platform and subjected to multi-modal *in vivo* PA imaging analysis employing a Vevo LAZR-X (FujiFilm VisualSonics Inc.)^43^ for high resolution anatomical and functional visualization of the implanted NETS. Anaesthesia was maintained at 2% throughout the imaging procedure. Photoacoustic (PA) imaging employs a non-ionizing laser (680–970 nm) to generate ultrasonic waves, which carry information around the stented artery. These waves were sensed by a transducer (MX250; 15–30 MHz) to generate ultrasound and photoacoustic images. The acquired images were then analyzed through Vevo LAB data analysis software.

#### (c) Induction of an in-stent thrombus and evaluation of photothermal thrombolysis

Thrombus formation was induced in the exposed rat common carotid artery (bearing the stent) by topical application of 10% FeCl_3_ for 5 min.^44–48^ Vascular injury due to oxidative damage of the endothelial cells triggers thrombus formation in the blood vessels. The stent was irradiated with an 808 nm NIR laser from an overhead laser source for 15 min, and the heat generated was captured with an infra-red thermal camera (FLIR). Occlusion of blood flow due to the growing thrombus and subsequent restoration of flow following NIR laser irradiation, as a measure of clot lysis, was assessed by placing a flow probe (1 PRB; PR-series, Transonic system) on the exposed common carotid artery.^49^ Signals from the artery were collected using a flow meter (Transonic, model T106) through a Doppler flow probe that measures the difference between the transit time of ultrasonic pulses propagating with and against the flow direction. Data were analysed using a PC-driven PowerLab data acquisition system employing LabChart Pro software (ADInstruments).

#### (d) Histology of stented carotid segments carrying thrombi

Stents carrying thrombi *in situ* were also subjected to histology to evaluate the extent of thrombolysis. Carotid arteries with implanted NETS, with or without laser irradiation, were harvested post-surgery from the rat and fixed in 4% paraformaldehyde for 8 to 12 h. Samples were dehydrated in 70% ethanol and infiltrated with the plastic embedding mixtures (MMA solution I, II and III) using a 3-step protocol in 15 ml Borosil glass vials as described.^50^ Samples were first dipped in solution I (60 ml methylmethacrylate, 35 ml butylmethacrylate, 5 ml methylbenzoate and 1.2 ml polyethylene glycol 400) on day 1, which was followed by incubation with solution II (100 ml solution I with 0.4 g dry benzoyl peroxide) on day 2. Finally, samples were infiltrated with solution III on day 3, which contains 100 ml solution I and 10.8 g dry benzoyl peroxide. Polymerization was initiated by adding 600 ml accelerator (*N*,*N*-dimethyl-*p*-toluidine) to 100 ml cold solution III followed by stirring, which was added to glass vials containing the infiltrated artery sample. As the polymerization process is sensitive to oxygen, glass vials were completely filled with the mixture and tightly capped to avoid oxygen contact. Next, the vials were transferred to a deep freezer for 3 days to complete the polymerization process.

After polymerization, plastic blocks were carefully removed from the glass vials and were trimmed to the desired size for sectioning. The 3–5 mm thick sections were cut using a rotary microtome (Leica RM2255) by keeping the block and blade moist with 30–40% methanol. Sections were transferred to slides coated with poly-l-lysine and carefully stretched or flattened using a rubber roller. Next, the slides were covered with polyethylene and clamped to a slide press, which was followed by incubation at 42 °C for 2 days. The de-plasticization of the sections was performed by employing two changes of dichloromethane and acetone for 20 and 10 min, respectively. Finally, sections were subjected to hematoxylin–eosin staining and analyzed under a light microscope (Nikon, model Eclipse Ti-E) at 4× magnification to evaluate the clot lysis efficacy of implanted NETS following NIR laser irradiation.

### Statistical methods

Data are presented as mean ± SEM of at least 3 independent experiments. Two-tailed Student's *t*-tests were performed using GraphPad Prism software to evaluate the significance in difference between groups. Values were considered significant when *p* < 0.05.

### Ethical statement

All animal procedures were carried out in strict adherence to the Guidelines for Care and Use of Laboratory Animals of Banaras Hindu University. The experimental protocols were approved by the Animal Ethics Committee of Banaras Hindu University (IAEC-3258).

## Results

### Characterization of coated thermogenic stents

**Fig. 1:**
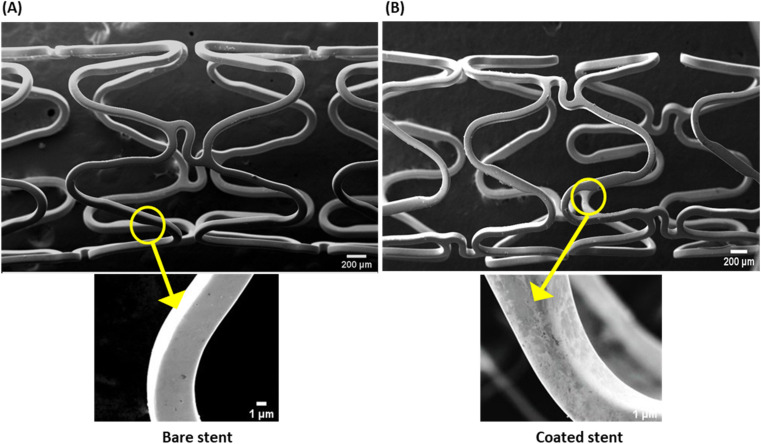
Scanning electron micrographs of surface morphologies of (A) bare (uncoated) and (B) coated stents. Magnifications: upper panels, 25× (scale, 200 μm); lower panels, 400× (scale, 1 μm).

**Fig. 2:**
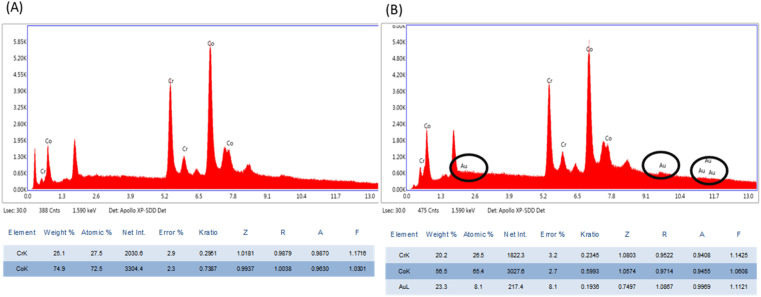
EDX analyses of (A) bare (uncoated) and (B) coated stents.

The thin plasmonic film of gold on the stent surface achieved by employing GNRs was characterized by SEM and EDX. The SEM images signified the rough surface associated with coated stents as compared to bare metal stents (Fig. 1), which was further examined by EDX analysis (Fig. 2). Chemical microanalysis of the coated stent confirmed the presence of an adequate quantity of the thin plasmonic film of gold on the thermogenic stent surface (Fig. 2). Raman spectra were used to examine the coating efficacy on the stent by assessing the structural and vibrational characteristics of gold. The image captured using a 50× magnification objective lens (Zeiss) of a Raman instrument showed a dense layer of coating on the stent surface (ESI Fig. 3A†). The observed prominent peak in the Raman spectrum clearly indicated the presence of gold.^51^ FTIR spectroscopy was performed to examine the coating on the stent surface. The spectrum of the coated stent exhibited characteristic peaks at ∼2930, 2850 and 1430 cm^−1^ that correspond to the presence of a thin plasmonic film of gold^52,53^ (ESI Fig. 3B†). It may be noted here that annealing at temperatures below 100 °C also yielded satisfactory results.

To investigate the stability of the glossy coating on the stent surface against hydrodynamic shear of perpetual intramural blood flow, the buffer was allowed to perfuse over the coated stents *in vitro* at the arterial shear rate (1500 s^−1^), which closely simulated fluidics *in vivo*.^32^ The buffer was allowed to flow over the stent for 6 to 8 h continuously each day for 30 days. The stability of the glossy coating was then verified from the uniformity in heat generated upon irradiation with the NIR laser on different days up to the 30th day. Temperature was recorded to be 60 °C, 61 °C, 58 °C and 59 °C, respectively, on days 0, 10, 20 and 30 (ESI Fig. 4†), which underscored the lack of erosion of the gold coating by hydrodynamic shear over time.

### Characterization of thrombolysis induced by the heat generated from NIR laser-irradiated thermogenic stents *in vitro*

**Fig. 3:**
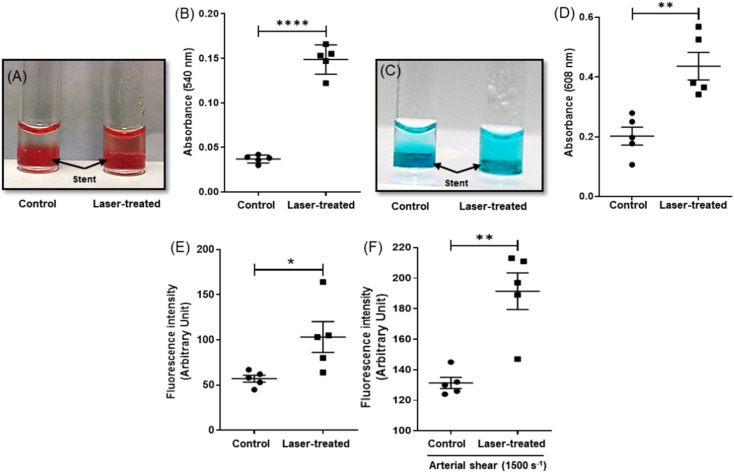
Photothermal ablation of in-stent/peri-stent clots exposed to the NIR laser for 30 min. Clots were generated by addition of 2–5 mM CaCl_2_ and 1 U ml^−1^ thrombin to a solution of fibrinogen (1 mg ml^−1^) carrying the thermogenic stents, followed by irradiation with the NIR (808 nm) laser at a power density 1.05 W cm^−2^. Whenever required, the fluorescent fibrin clot was generated by addition of Alexa Fluor 488 – conjugated fibrinogen (10% v/v) to the above solution. Drabkin's assay (A) and (B) and methylene blue assay (C) and (D) were carried out in order to evaluate the extent of clot lysis. (E) In-stent or peri-stent clot lysis analyzed employing an Alexa Fluor 488-labeled thrombus. (F) Lysis of a fluorescently labeled stent thrombus exposed to arterial hydrodynamic shear (1500 s^−1^). Data are representative of five different sets of experiments (mean ± SEM). *P < 0.05; **P < 0.01; ****P < 0.0001, *vs.* control.

In order to study the efficiency of NETS in inducing photothermal ablation of clots, a thrombus was allowed to form around the stent placed within a glass cuvette followed by irradiation with the NIR laser for 30 min, which raised the temperature up to 50 °C. The extent of thrombolysis was evaluated by three standard assays as described earlier.^32^ Photothermal fibrinolysis was found to be 57% higher in the fluorescence-based assay whereas the rise was 73% each in Drabkin's and methylene blue assays, respectively (Fig. 3A–E). In order to evaluate stent thrombolysis under arterial hydrodynamic shear resembling the physiological milieu, the fluid was allowed to perfuse over the stent carrying a fluorescent thrombus at a shear rate of 1500 s^−1^ (ESI Fig. 2†). The extent of thrombolysis as quantified from the release of Alexa 488-labeled fibrin was found to be 35% (Fig. 3F), which thus confirmed photothermal ablation of stent thrombosis.

The heat-induced alteration in fibrin clot structural morphology following 30 min NIR laser irradiation was evaluated using orthogonal tools like SEM and CLSM. The SEM images were consistent with a significant 12-fold increase in thrombus-depleted areas (ESI Fig. 5A–C†) associated with a drop (by 5%) in fibrin diameter (ESI Fig. 5D and E†). In agreement, confocal images too demonstrated a nearly 5 times expansion in areas of thrombus depletion examined at different depths in Z-stacking mode (ESI Fig. 6A–C†) and 56% reduction in fiber density (ESI Fig. 6D†) in laser-irradiated samples.

**Fig. 4:**
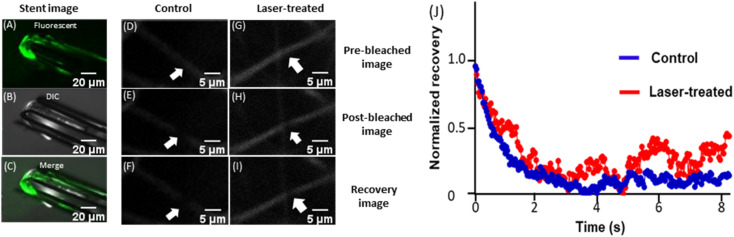
FRAP analysis of a laser irradiated in-stent thrombus. (A–C) Confocal images of an in-stent fluorescent clot (10×) representing fluorescent (A), differential interference contrast (DIC) (B) and merged (C) images. (D–I) Pre-bleached, post-bleached and fluorescence recovery images (63×) of control and NIR laser-irradiated samples, as stated. Arrows indicate the regions of interest (ROI) on the fluorescent thrombus. (J) Kinetics of fluorescence recovery in control and NIR laser-treated samples.

Thermal destabilization of in-stent/peri-stent thrombi following 15 min NIR-laser exposure was further validated employing FRAP analysis. FRAP characterizes the dynamism of fibrin monomers/oligomers within the clot by quantifying fibrin mobility and turnover kinetics.^32^ The in-stent clot was focused on and observed under a microscope (Fig. 4A–C). A narrow region within the fluorescent thrombus was photobleached using a 488 nm laser (at 100% power) and fluorescence recovery in that region was recorded over time at 2% excitation laser power in both control (Fig. 4D–F), as well as NIR laser-irradiated (Fig. 4G–I) thrombi. The speed of fluorescence recovery in thermally loosened fibrin strands was reflective of the extent of fibrin mobility within destabilized thrombi, which amounted to 13.4% mobile fibrin fractions in NIR laser-irradiated stents as compared to 5.3% in non-irradiated counterparts (ESI Fig. 7†). Thus, results from this study underscore enhanced fibrin dynamism within the in-stent thrombi following photothermal heat generation that could lead to thrombus rarefication.

### Photothermal lysis of in-stent thrombi and partial restoration of blood flow upon NIR laser irradiation of implanted NETS

**Fig. 5:**
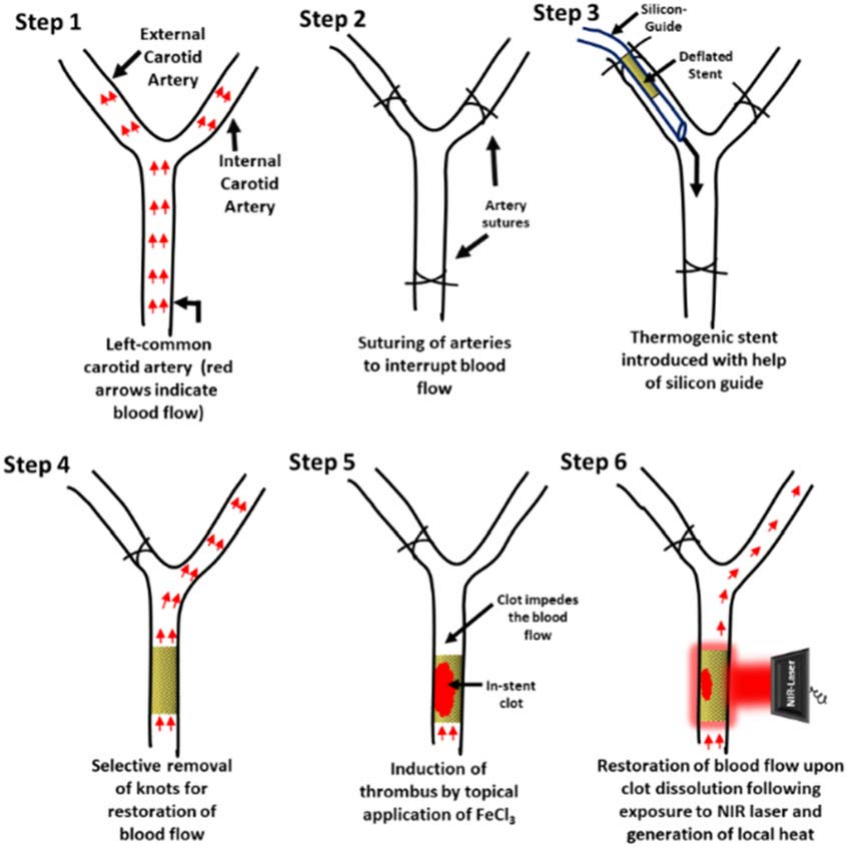
Scheme for grafting a thermogenic stent in the common carotid artery of a rat, induction of a thrombus and photothermal thrombolysis.

**Fig. 6:**
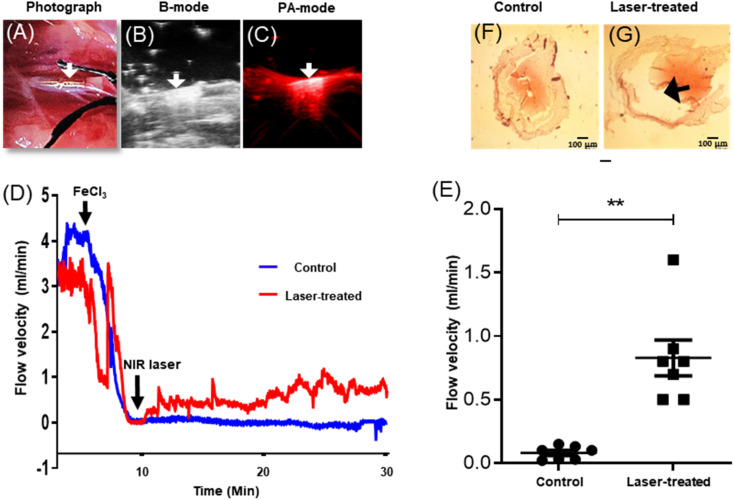
Photothermal lysis of an in-stent thrombus and restoration of the blood flow. (A) Photograph of NETS implanted in the rat common carotid artery; (B) and (C) photoacoustic imaging of implanted NETS in B-mode (ultrasound) and PA-mode (photoacoustic), respectively. The arrow indicates the position of the stent. (D) Measurement of blood flow velocity in the rat common carotid artery with the implanted stent with a flow meter showing occlusion of blood flow due to the growing thrombus and subsequent restoration of flow following NIR laser irradiation. (E) Scatter dot plots representing the corresponding flow velocities after 15–20 min irradiation with the NIR laser or controls without laser exposure (*n* = 7 in each group). Each dot in the scatter plots represents an independent observation. **P < 0.01 *vs.* control. (F) and (G) Light microscopy of hematoxylin- and eosin-stained transverse sections of stented carotid segments harvested post-surgery from sacrificed animals with or without irradiation with the NIR laser (the arrow indicates reduction in thrombus size; scale, 100 μm).

Rats medicated with oral aspirin (10 mg kg^−1^ d^−1^) for 5 days^42^ were anesthetised. The coated thermogenic stents (length, 2.5 mm; diameter, 635 μm; strut thickness, 70 μm) were carefully guided into and deployed in the murine common carotid artery (Fig. 5 and 6A), as described. The anatomical and optoacoustic imaging of the implanted stent was carried out in B (ultrasound)-mode and PA (photoacoustic)-mode by employing a photoacoustic imaging platform (Fig. 6B and C). The thrombus was induced locally by topical application of 10% FeCl_3_, which was followed by irradiation with a 3 W solid-state continuous diode laser (808 nm) for 15 min. Temperature around the stent was found to be elevated up to 47 °C following laser exposure as captured by a thermal camera. Blood flow was assessed by placing a Doppler flow probe on the exposed artery, the signal from which was collected through a transonic flow probe and further analysed. Restriction in blood flow due to the growing thrombus was found to be 98%, which was partially restored (by 30%) upon laser irradiation (Fig. 6D and E), underscoring the applicability of the novel NETS as an essential tool against stent-thrombosis. The hematoxylin–eosin-stained sections of stented carotid segments revealed a significant reduction in size of the in-stent thrombus in laser-treated rats as compared to control animals (Fig. 6F and G; ESI Fig. 8†), thus validating the therapeutic efficacy of NETS.

## Discussion

In this report we demonstrate the potential of ‘thermogenic’ stents (NETS) in bringing about relief from ‘stent thrombosis’, a life-threatening complication that warrants urgent interventions like repeat angioplasty and/or CABG surgery. Notably, none of the existing stent technologies permits lysis of the in-stent thrombus, thus underlining the need for novel approaches to counter the threat of scaffold thrombosis. Subjects carrying coated NETS can be irradiated frontally from an external NIR laser source upon diagnosis of stent thrombosis. The heat generated from the deployed stent would result in ablation/rarefication of the thrombus in and around the implant leading to restoration of blood flow that can be monitored with color Doppler. Although we have observed ∼30% restoration in blood flow following 15 min irradiation with the laser, the duration of laser exposure can be expanded to achieve greater relief from occlusion. The occluded stent can thus become functional again in the absence of any painful invasive procedure or expensive setup and the patient can be discharged early. A coating of a NIR-active material on the stent surface was found to be stable against fluid shear and can coexist with the drug-eluting polymer.

Photothermal therapy (PTT) is a well-established but burgeoning field of medical research that has been widely employed in wound healing,^14–17^ treatment of bacterial infection,^18–21^ pain relief,^22,23^ drug delivery^24–27^ and for selective thermal ablation of malignant tissues^54–58^ at temperatures above 40 °C, which ensues protein denaturation and disruption of target cell membranes. Subsequent studies from our group and others established the anti-thrombotic attributes of PTT as it leads to disintegration of fibrin-rich clots by segregation of component fibrin strands, which are then washed off from the lesion site under arterial hydrodynamic shear.^32–35^ These studies opened newer areas of application for thermal therapy against mural thrombosis in addition to its well reported application against tumors.

The effect of temperature on tissues surrounding the deployed stent is the subject of future investigation. Temperature at the ‘heat focus’ area (maximum ∼47 °C) is significantly quenched by the ongoing blood flow (2 mm s^−1^), thus restricting the temperature in the surrounding blood from exceeding ∼43 °C.^59^ Temperatures in the intimal layer and sub-endothelium in direct contact with the stent drop nonlinearly with depth that is further compensated by the blood flow.^59^ Considering the thickness of intima to be ∼600 μm,^60^ conduction of heat to cardiac tissues is tolerated without seriously compromising cell viability and primarily localized to the intima.

Although ours is the first study directed at photothermal management of stent thrombosis, hyperthermia has earlier been employed for control of in-stent restenosis as mild heat (between 45 and 50 °C) effectively destroys intimal cells.^61–63^ Hyperthermia, too, has successfully been used for controlled site-specific drug delivery around the implanted magnetizable stent when applied under a uniform magnetic field.^64^ There have been several approaches in the past to expose the stent *in situ* to local heat though each procedure has its share of limitations. These include invasive procedures like insertion of a special thermal catheter into the deployed stent,^65^ or guiding the NIR beam through the optical fiber to endovascular locations,^59,66,67^ as well as non-invasive methods like induction (eddy-current) heating of stents using electromagnetic fields.^62,68,69^ Non-invasive attempts have been made like induction (eddy-current) heating of stents using electromagnetic fields,^62^ or forming a radiofrequency resonant circuit.^70^ PTT and photothermal effect-induced drug release have also been explored previously as a modality to prevent in-stent restenosis by employing gold nanoparticle-coated multifunctional stents.^59^

The penetration depth of the NIR laser at 808 nm is about 2 cm,^36,69^ which can be further enhanced by switching to longer wavelength NIR (1000–1350 nm; bio window II),^57,71^ thus allowing non-invasive photothermal ablation of stent thrombosis when irradiated from an external source. Alternatively, the occluded stent can be irradiated with an optical fiber-guided NIR laser^72,73^ though guiding the NIR beam to endovascular locations for PTT still remains challenging. In conclusion, this study for the first time presents a proof-of-concept in favour of deployment of thermogenic coronary scaffolds as a guard against stent thrombosis and requires further investigation towards clinical validation.

## Data availability

All relevant data that support the findings of this study are provided within the paper. Raw data incorporated in Microsoft excel and/or GraphPad Prism files are available on request from the corresponding authors.

## Author contributions

D. D. supervised the entire work; N. S., P. P. K. and P. T. performed various experiments; D. D., V. A., N. S. and P. P. K. analyzed the results and wrote the manuscript.

## Conflicts of interest

The authors declare no competing interests.

## Supplementary Material

NA-006-D3NA00751K-s001
