# Supplementary material for: Nanogold-coated stent facilitated non-invasive photothermal ablation of stent thrombosis and restoration of blood flow
Source: Nanoscale Adv. 2024 Jan 30;6(5):1497–506. doi: 10.1039/d3na00751k (PMC10898437; doi:10.1039/d3na00751k)
Supplement: NA-006-D3NA00751K-s001 [file NA-006-D3NA00751K-s001.pdf]

Electronic Supplementary Material (ESI) for Nanoscale Advances.
This journal is © The Royal Society of Chemistry 2024

Supplementary information:

Materials and Methods:

Raman scattering: The Raman spectra of the thermogenic stent were acquired using a Witec Alpha 300 RAS microspectrometer with excitation wavelengths of 532 nm and 633 nm, generated by an Ar+ laser and a semiconductor laser, respectively [52]. Raman measurements were conducted on the Raman microscope (WITec) with an excitation wavelength of 785 nm and a detection range of 100–4000 cm^{-1}. Spectra were collected using a 50X magnification objective lens (Zeiss), and data acquisition involved an integration time of 30s and 2 accumulations. The acquired spectra were processed using WITec Project FOUR and GraphPad Prism software.

Fourier-transform infrared spectroscopy (FTIR): ATR-FTIR spectra of coated thermogenic stent were obtained using a Nicolet 6700 FTIR spectrometer (Thermo Fisher Scientific) equipped with a GladiATR module (PIKE Technologies) and a diamond crystal with a contact area of 0.5 mm^2 [53, 54]. Spectra were recorded in the 4000–400 cm^{-1} range with a 4 cm^{-1} resolution and atmospheric correction switched on at room temperature (25°C). Spectra were generated using OMNIC software as a mean of 34 scans from which the background spectrum was subtracted and further analyzed using Origin 2016 software.

Scanning electron microscopy: Fibrin clot was generated around the thermogenic stent and subjected to NIR irradiation for 30 min as described. Both control (no laser) and irradiated samples were fixed with glutaraldehyde/paraformaldehyde mix (1% each) for 24 h, followed by washing sequentially with 1X PBS and distilled water. Samples were frozen at −80°C for 3-4 h, lyophilized in a freeze-dryer for 6-8 h at -55 °C and desiccated prior to SEM analysis. Sputtering was carried out on mounted samples employing SEM sputter coater (Quorum Technologies, model SC7620)**,** and change in surface morphology of fibrin clot was recorded using an analytical SEM (Zeiss, model EVO 18) in the high vacuum mode at an accelerating voltage of 10 kV. The diameter of the fibrin fibres and the thrombus-depleted areas were determined employing Image J analysis software. Samples were prepared in triplicate and 60 individual fibre diameters were evaluated from each SEM image.

Confocal laser scanning microscopy: Fluorescently labelled clot was generated around thermogenic stent using Alexa Fluor 488–conjugated fibrinogen, followed by exposure to NIR laser for 30 min. Samples were subjected to confocal microscopy (Zeiss, model LSM 700) in Z-stacking mode to analyse the 3-dimensional structure of fibrin clots at different depths. A 63X oil immersion objective with 1 AU pinhole size was employed to capture 3D images (excitation, 488 nm; emission, 510 nm) using Zen imaging software. Fiber density (measured between two-parallel horizontal lines)

and thrombus-depleted areas in control and irradiated samples were analysed in single optical sections of different images using the Image J software and were plotted using GraphPad Prism software.

Figures:

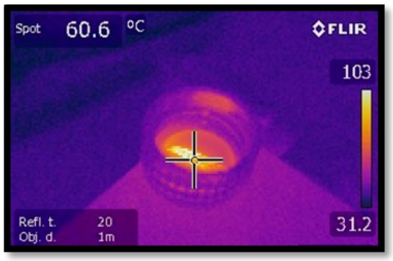

Supplementary Figure 1. Infra-red thermal image of coated stent irradiated with 808 nm NIR laser. The cursor within the panel represents spot temperature and the vertical pseudo-color bar signifies temperature intensity from high (yellow) to low (dark blue).

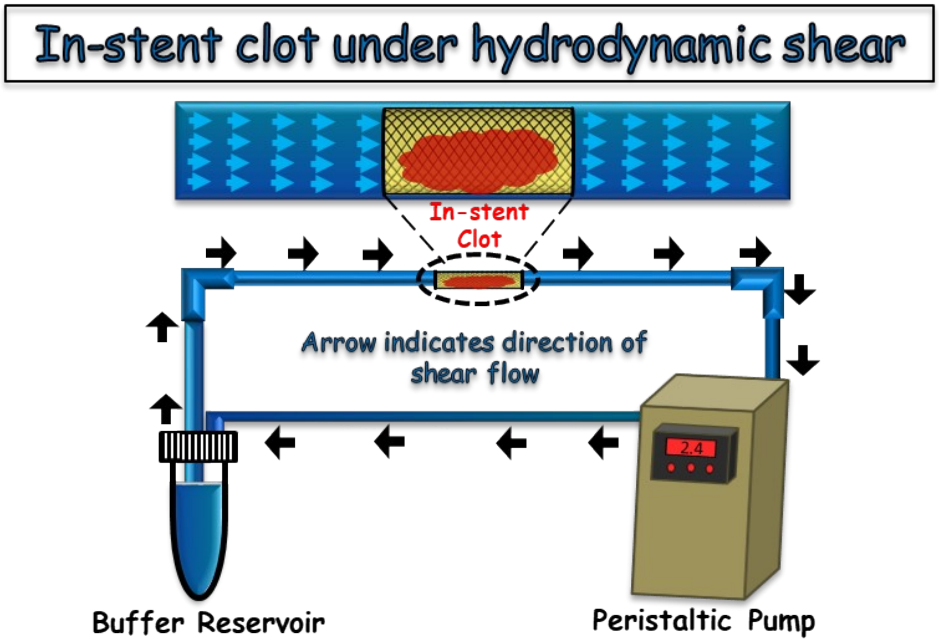

Supplementary Figure 2. Experimental set-up for the study of in-stent thrombolysis against arterial shear (1500 s^{-1}) under dynamic flow conditions.

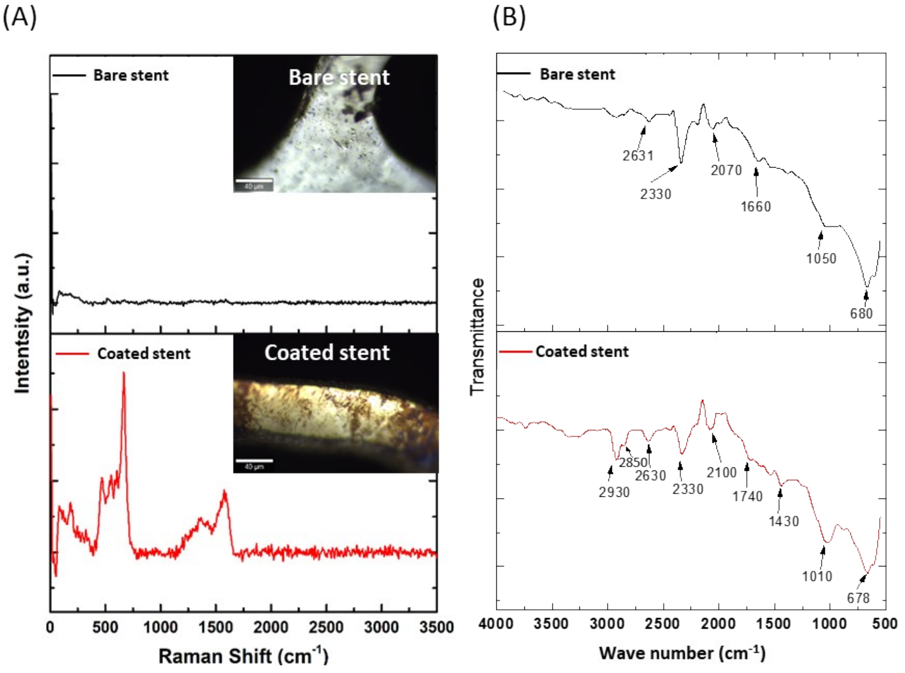

Supplementary Figure 3. Raman spectra (panel A) and FTIR spectra (panel B) of bare metal (black) and coated (red) stents (scale, 40 µm).

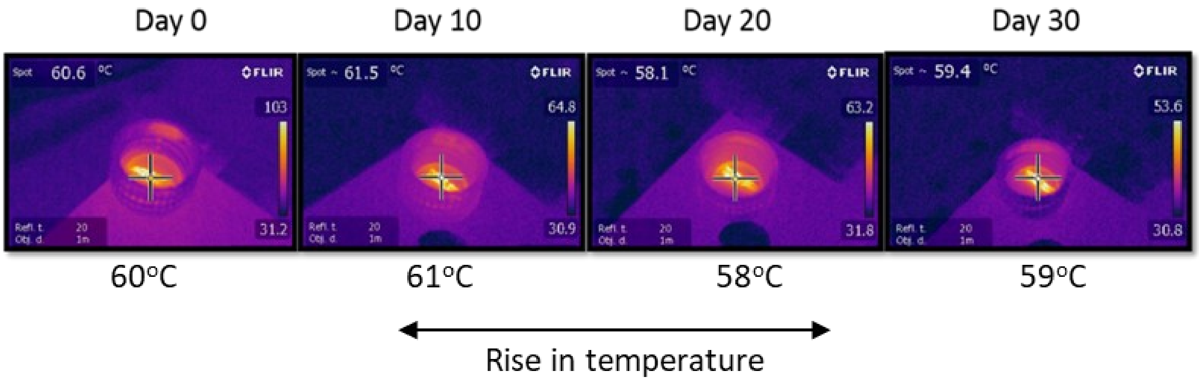

Supplementary Figure 4. Infra-red thermal images of coated stent subjected to hydrodynamic shear at 1500 s^{-1} (arterial shear) from 0 to 30 days. Within each panel, the cursor represents spot temperature and the vertical pseudo-color bar signifies temperature intensity from high (yellow) to low (dark blue).

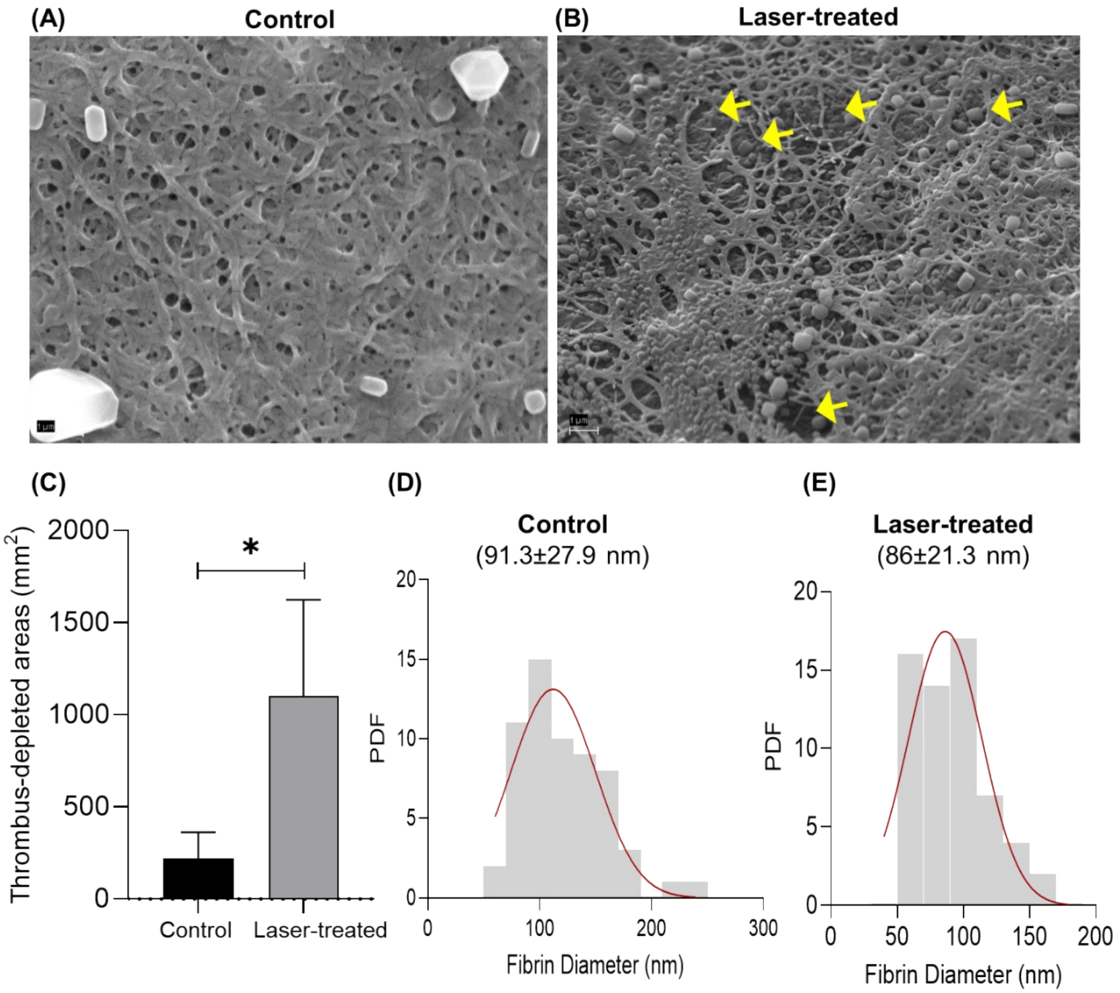

Supplementary Figure 5. Scanning electron microscopy of in-stent / peri-stent clots exposed to NIR laser for 30 min. Clots were generated by adding 2·5 mM $CaCl_2$ and 1 U/ml thrombin to solution of fibrinogen (1 mg/ml) carrying the thermogenic stents, followed by irradiation with NIR (808 nm) laser at power density 1.05 W/cm2. A and B, SEM images of clots in control and laser-irradiated samples (scale, 1 μm) exhibiting significant rarefication of clots upon laser irradiation (arrows indicating thrombus-depleted areas). C, bar diagrams representing thrombus-depleted areas in control and irradiated samples analyzed. D and E, fibrin diameter calculated from 60 fibers in each image from control and laser-treated samples (PDF, probability density function of the distribution). Data are representative of three different sets of experiments (mean ± SEM). *P < 0.05 vs Control.

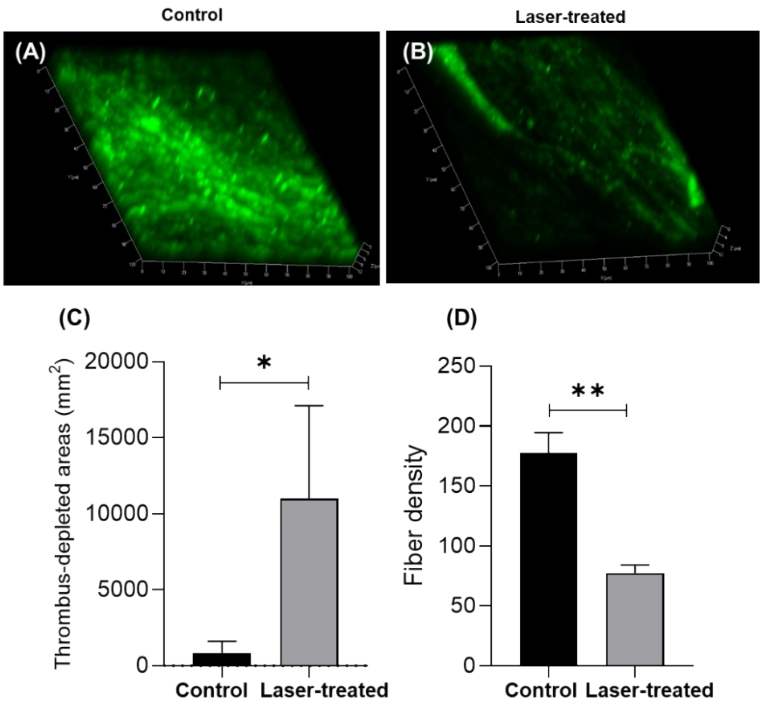

Supplementary Figure 6. Confocal microscopy of in-stent / peri-stent fluorescently labelled fibrin clots subjected to NIR laser irradiation. A and B, z-stack (6-16 µm) images of control and laser-treated samples exhibiting upon laser irradiation. C, bar diagrams representing thrombus-depleted areas in control and irradiated samples. D, fibrin fiber density was calculated in control and irradiated samples by counting fibers present within two horizontal lines drawn using Image J software. Data are representative of three different sets of experiments (mean ± SEM). *P < 0.05 vs Control.

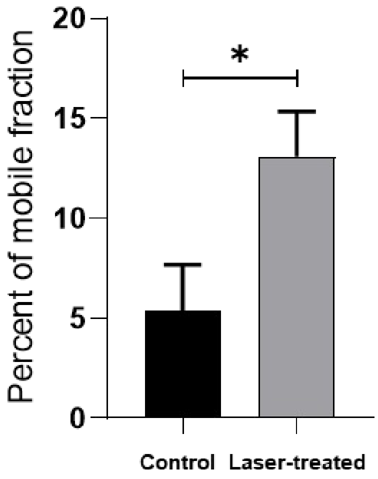

Supplementary Figure 7. FRAP analysis of laser-irradiated in-stent clot. Change in percent of mobile fraction in control vs NIR laser-treated samples. Data are representative of three different sets of experiments (mean ± SEM). *P < 0.05 vs Control.

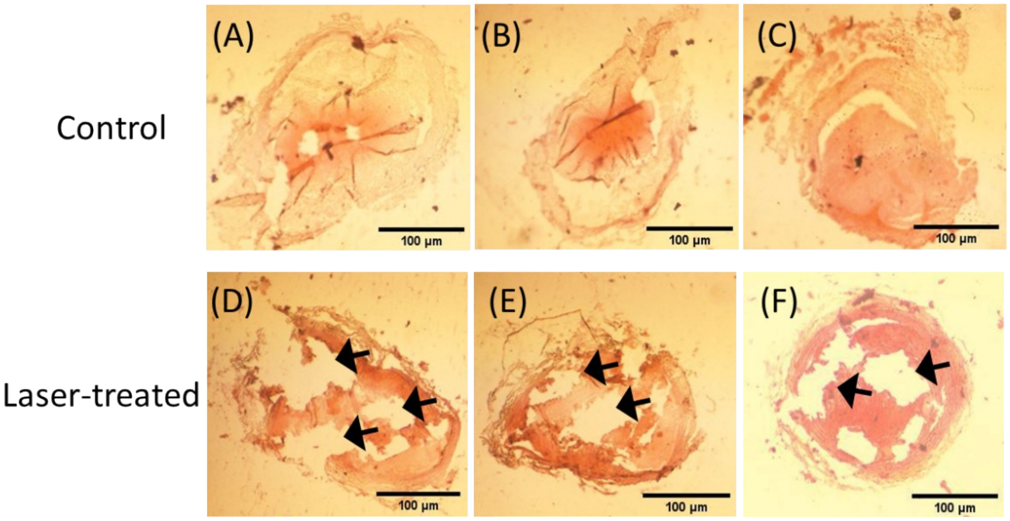

Supplementary Figure 8. Light microscopy images of hematoxylin- and eosin- stained transverse sections of stented carotid segments harvested post-surgery from control (A, B, and C) and NIR laser-irradiated (D, E, and F) animals (arrows indicating thrombus-depleted areas). Data are representative of three different sets of experiments.
